# Are There Any Differences in the Prognostic Value of Left Ventricular Ejection Fraction in Coronary Artery Disease Patients With or Without Moderate and Severe Mitral Regurgitation?

**DOI:** 10.3389/fcvm.2022.799253

**Published:** 2022-03-04

**Authors:** Qiang Li, Yifei Zhang, Haozhang Huang, Weihua Chen, Shanshan Shi, Shiqun Chen, Bo Wang, Wenguang Lai, Zhidong Huang, Zhiling Luo, Jiyan Chen, Ning Tan, Jin Liu, Yong Liu

**Affiliations:** ^1^Department of Cardiology, Guangdong Provincial Key Laboratory of Coronary Heart Disease Prevention, Guangdong Cardiovascular Institute, Guangdong Provincial People's Hospital, Guangdong Academy of Medical Sciences, Guangzhou, China; ^2^Fuwai Yunnan Cardiovascular Hospital, Kunming, China; ^3^The Second School of Clinical Medicine, Southern Medical University, Guangzhou, China; ^4^Longyan First Affiliated Hospital of Fujian Medical University, Longyan, China; ^5^The Third Clinical Medicine College, Fujian Medical University, Fuzhou, China; ^6^Guangdong Provincial People's Hospital, School of Medicine, South China University of Technology, Guangzhou, China

**Keywords:** left ventricular ejection fraction, prognosis, coronary artery disease, mitral regurgitation, indicator

## Abstract

**Background:**

Left ventricular ejection fraction (LVEF) is a vital variable to describe left ventricle systolic function and contractility of left ventricle. However, the association between LVEF and the prognostic effect in patients with moderate or severe mitral regurgitation (MR) is still controversial.

**Methods:**

This study comprised 30,775 coronary artery disease (CAD) patients who underwent coronary arteriography (CAG) in the Cardiorenal ImprovemeNt (CIN) registry from January 2007 to December 2018. Patients were divided into none or mild MR group and moderate or severe MR group, and 3 levels of LVEF ≥50, 40–50%, and <40% were further distinguished according to hospital baseline. Univariate and multivariate Cox proportional analyses were used to investigate the association between LVEF levels and long-term all-cause mortality in patients with different MR severities.

**Results:**

Of 30,775 CAD patients (62.9 ± 10.6 years, females 23.8%), 26,474 (86.0%) patients had none or mild MR. Compared with none or mild MR patients, patients with moderate or severe MR were older and had worse cardio-renal function. In multivariable Cox proportional analysis, LVEF <40% was independently associated with higher mortality compared with LVEF ≥ 50% in all kinds of MR severity {none or mild MR [adjusted hazard ratio (HR): 1.79; 95% CI: 1.56–2.05, *p* < 0.001], moderate or severe MR [adjusted HR: 1.57; 95% CI: 1.29–1.91, *p* < 0.001]}.

**Conclusions:**

LVEF is a reliable prognostic index in CAD patients, even in those with moderate or severe MR. LVEF monitoring would still be clinically useful in CAD patients with moderate or severe MR. Clinical trials are needed to prospectively evaluate the optimal threshold for LVEF in patients with moderate or severe MR.

## Introduction

Atherosclerotic coronary artery disease (CAD) remains a major health burden globally and is a leading cause of morbidity and mortality worldwide (1). Myocardial ischemia affects patient's cardiac function and leads to a decrease in left ventricular ejection fraction (LVEF). Therefore, LVEF is considered to be an important indicator of cardiac function and prognosis in patients with CAD (2).

Mitral regurgitation (MR) is a growing public health problem, which generally progresses insidiously, and causes left-ventricular overload and dysfunction (3). Some studies have shown that nearly one in five CAD patients have MR (4), and MR significantly increases the risk of mortality in patients (5–7). However, moderate or severe MR increases the actual measurement of LVEF and overestimates patient cardiac function. In patients with CAD combined with moderate or severe MR, the relationship between LVEF and prognosis is unclear.

Our primary objective was to investigate the association between LVEF and long-term prognosis in CAD patients with different severities of MR.

## Methods

### Study Design and Patient Selection

The Cardiorenal ImprovemeNt (CIN) Registry is a single-center, observational cohort study. During the period from January 2007 to December 2018, a total of 88,938 patients underwent coronary arteriography (CAG) and 59,667 patients were diagnosed with CAD according to the 10th Revision Codes of the International Classification of Diseases (ICD-10; I20.xx–I25.xx, I50.00001, and I91.40001) in Guangdong Provincial People's Hospital, China (ClinicalTrials.gov NCT04407936).

We excluded participants aged <18 years (*n* = 19), with cancer (*n* = 879), with missing echocardiographic data (*n* = 12,988), who underwent CAG followed by mitral valve operation (*n* = 481), and who lacked follow-up LVEF data (*n* = 14,525). Finally, 30,775 CAD patients were included in our study (Supplementary Figure 1).

### Data Extraction

The presence of MR and the levels of LVEF were confirmed by the first examination of echocardiography. All CAD patients were stratified into 2 groups based on MR severity (none or mild MR vs. moderate or severe MR). MR severity was mainly evaluated by visual assessment integrating Doppler data from multiple acoustic windows, incorporating qualitative and semi-quantitative methods (8, 9).

The calculations for LVEF used the biplane-Simpson method by the end diastolic and end systolic apical 4- and 2-chamber views. Patients were divided based on MR severity into 3 groups according to the classification of the American College of Cardiology (ACC) as follows: normal: LVEF ≥ 50%; mild dysfunction: 40% ≤ LVEF <50%; moderate or severe dysfunction: LVEF <40%. In addition, the data quality control and periodical database verification were controlled by senior echocardiography physicians.

### Outcomes and Definitions

The endpoint of the study was long-term all-cause mortality. Patient's follow-up information was obtained from the Guangdong Provincial Public Security, which was matched with the electronic Clinical Management System of the Guangdong Provincial People's Hospital records according to the unique ID number of patients.

The comorbidities involved: hypertension (HT); diabetes mellitus (DM); acute myocardial infarction (AMI); congestive heart failure (CHF), defined as New York Heart Association class >2 or Killip class >1 (10); atrial fibrillation (AF); stroke; chronic kidney disease (CKD), defined as eGFR ≤ 60 ml/min/1.73 m^2^; anemia, defined as hematocrit <36% for women and <39% for men (11); hyperlipidemia, defined according to 2016 ESC guidelines for treating dyslipidemias (12).

### Statistical Analysis

All results were summarized and stratified into 2 groups according to MR severity (none or mild MR vs. moderate or severe MR). Descriptive statistics are reported as the mean [standard deviation (SD)], median [interquartile range (IQR)], or number and percentage when appropriate. Continuous variables were tested for differences between groups using *t*-test and ANOVA, and Pearson chi-squared tests for dichotomous variables, using Fisher's exact test when needed. Time-to-event data were presented graphically using Kaplan–Meier curves. Log-rank tests were used to compare the survival rate among LVEF subgroups.

The association between LVEF and log-term mortality was assessed by univariate and multivariate Cox proportional analyses in different models. Model 1 involved univariate Cox analysis, model 2 involved adjustment of age (as a continuous variable) and gender, and model 3 involved adjustment of demographic characteristics (age and gender), complications [HT, DM, AMI, CHF, CKD, AF, stroke, anemia, percutaneous coronary intervention (PCI), and hyperlipidemia], and medications [angiotensin-converting enzyme inhibitor/angiotensin receptor blocker (ACEI/ARB), beta-blockers, calcium channel blocker (CCB), statins, antiplatelet, mineralocorticoid receptor antagonist (MRA), loop diuretics, and oral anticoagulants (OAC)]. A value of *p* < 0.05 was considered statistically significant, and all statistical tests were two-sided. All statistical analyses were undertaken using R 4.0.3 (R Institute for Statistical Computing, Vienna, Austria).

## Results

### Baseline Characteristics

We analyzed 30,775 patients with CAD, who were diagnosed from January 2007 to December 2018 [mean age 62.9 ± 10.6 years, 7,328 (23.8%) females]. In total, 6,340 (20.6%) patients had AMI, 3,057 (9.9%) had CHF, 17,350 (56.4%) had HT, and 973 (3.2%) had AF. Patients were divided into 2 groups according to MR severity; 26,474 (86.0%) patients had none or mild MR, and 4,301 (14.0%) patients had moderate or severe MR.

Compared to patients with none or mild MR, those with moderate or severe MR were older, had higher pro-brain natriuretic peptide (pro-BNP), a larger left ventricular end-diastolic dimension (LVEDD), and lower LVEF. In contrast, patients in moderate or severe MR were more likely to combine with AMI, CHF, AF, DM, CKD, anemia, hyperlipidemia, and were less likely to use HT, beta-blockers and CCB. The detailed clinical characteristics of patients are listed in Table 1.

**Table 1:** Baseline characteristics according to categories of mitral regurgitation (MR).

**Characteristic**	**Overall**	**None or Mild MR**	**Moderate or Severe MR**	***P*-value**
	**(*n* = 30,775)**	**(*n* = 26,474)**	**(*n* = 4,301)**	
**Demographic**				
Female, *n* (%)	7,328 (23.8)	6,257 (23.6)	1,071 (24.9)	0.074
Age, years	62.90 (10.57)	62.62 (10.59)	64.60 (10.31)	<0.001
Medical insurance, *n* (%)				
Self-paying	4,792 (15.6)	4,196 (15.8)	596 (13.9)	<0.001
Urban insurance	21,078 (68.5)	18,042 (68.1)	3,036 (70.6)	
Rural insurance	1,307 (4.2)	1,162 (4.4)	145 (3.4)	
Other	3,598 (11.7)	3,074 (11.6)	524 (12.2)	
**Comorbidities**				
AMI, *n* (%)	6,340 (20.6)	5,316 (20.1)	1,024 (23.8)	<0.001
Anemia, *n* (%)	9,739 (33.0)	8,053 (31.7)	1,686 (40.6)	<0.001
CHF, *n* (%)	3,057 (9.9)	2,165 (8.2)	892 (20.7)	<0.001
HT, *n* (%)	17,350 (56.4)	14,952 (56.5)	2,398 (55.8)	0.384
DM, *n* (%)	8,300 (27.0)	6,941 (26.2)	1,359 (31.6)	<0.001
CKD, *n* (%)	5,603 (18.2)	4,365 (16.5)	1,238 (28.8)	<0.001
Hyperlipidemia, *n* (%)	19,803 (66.4)	16,990 (66.2)	2,813 (67.8)	0.044
AF, *n* (%)	973 (3.2)	633 (2.4)	340 (7.9)	<0.001
COPD, *n* (%)	256 (0.8)	214 (0.8)	42 (1.0)	0.300
Stroke, *n* (%)	1,748 (5.7)	1,472 (5.6)	276 (6.4)	0.027
PCI, *n* (%)	22,233 (72.2)	19,298 (72.9)	2,935 (68.2)	<0.001
**Laboratory test**				
LDL-C, mmol/L	2.83 (0.98)	2.82 (0.98)	2.86 (0.96)	0.046
HDL-C, mmol/L	1.00 (0.26)	1.00 (0.26)	0.97 (0.26)	<0.001
eGFR, ml/min/1.73 m^2^	77.14 (24.72)	78.38 (24.38)	69.87 (25.44)	<0.001
ALB, g/L	36.27 (4.23)	36.48 (4.12)	35.03 (4.68)	<0.001
pro-BNP, pg/ml^⋇^	271.80 [71.85, 1108.50]	214.00 [63.18, 834.20]	1242.00 [307.28, 3357.25]	<0.001
**Echocardiography**				
LVEF, %	58.92 (12.08)	60.30 (10.99)	50.45 (14.71)	<0.001
LVEDD, mm	48.51 (7.62)	47.67 (7.08)	53.70 (8.68)	<0.001
LVESD, mm	32.10 (8.42)	31.00 (7.36)	38.89 (10.95)	<0.001
**Medication**				
ACEI/ARB, *n* (%)	15,452 (51.0)	13,363 (51.1)	2,089 (50.0)	0.180
Beta-blockers, *n* (%)	24,741 (81.6)	21,437 (82.0)	3,304 (79.1)	<0.001
CCB, *n* (%)	6,250 (20.6)	5,509 (21.1)	741 (17.7)	<0.001
Statins, *n* (%)	28,649 (94.5)	24,820 (95.0)	3,829 (91.7)	<0.001
Antiplatelet, *n* (%)	28,913 (95.4)	25,084 (96.0)	3,829 (91.7)	<0.001
Loop diuretics, *n* (%)	4,518 (14.9)	2,980 (11.4)	1,538 (36.8)	<0.001
MRA, *n* (%)	4,672 (15.4)	3,106 (11.9)	1,566 (37.5)	<0.001
OAC, *n* (%)	1,285 (4.2)	838 (3.2)	447 (10.7)	<0.001

⋇ *median expression. MR, mitral regurgitation; AMI, acute myocardial infarction; CHF, congestive heart failure; HT, hypertension; DM, diabetes mellitus; CKD, chronic kidney disease; AF, atrial fibrillation; COPD, chronic obstructive pulmonary disease; PCI, percutaneous coronary intervention; LDL-C, low-density lipoprotein cholesterol; HDL-C, high-density lipoprotein cholesterol; eGFR, estimated glomerular filtration rate; ALB, albumin; pro-BNP, pro-brain natriuretic peptide; LVEF, left ventricular ejection fraction; LVEDD, left ventricular end-diastolic dimension; LVESD, left ventricular end systolic diameter; ACEI/ARB, angiotensin-converting enzyme inhibitor/angiotensin receptor blocker; CCB, calcium channel blocker; MRA, mineralocorticoid receptor antagonist; OAC, oral anticoagulants*.

### Association of the Baseline LVEF With the Risk of Mortality

**Figure 1:**
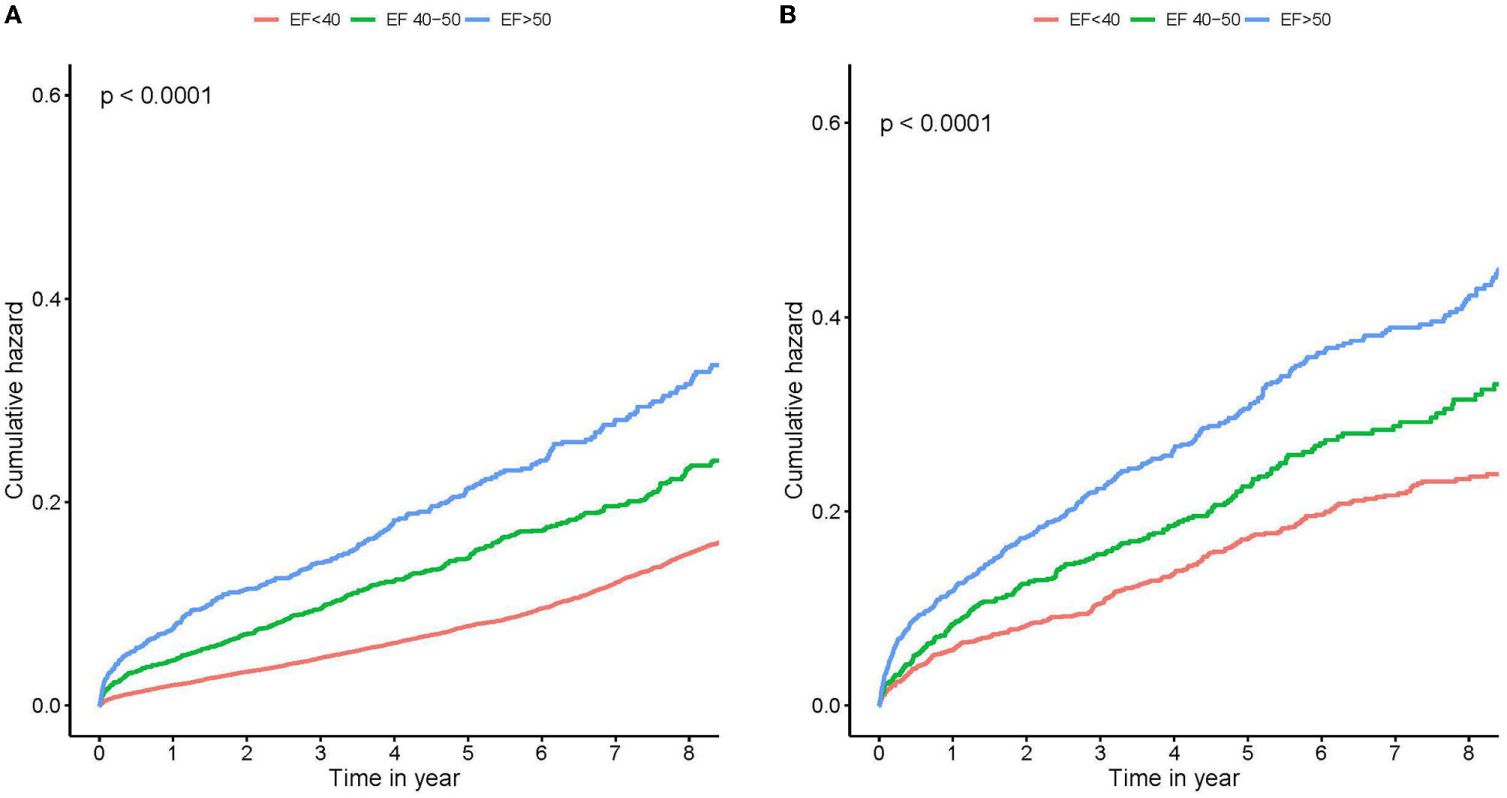
Kaplan–Meier survival curves stratified by left ventricular ejection fraction (LVEF). Survival stratified by LVEF <40, 40 to 50%, and ≥50% throughout none or mild MR **(A)**, and moderate or severe MR **(B)**. Note the large mortality difference between different LVEF groups. MR, mitral regurgitation.

We examined the prognostic effects of different levels of LVEF in patients with CAD combined with different degrees of MR. In none or mild MR group, Kaplan–Meier curves showed that patients in the group with lower baseline mean LVEF had a higher risk of all-cause death than patients in the other groups during the follow-up period (log-rank test, *p* < 0.001; Figure 1A). Similar results were obtained in patients with moderate or severe MR (log-rank test, *p* < 0.001; Figure 1B).

**Figure 2:**
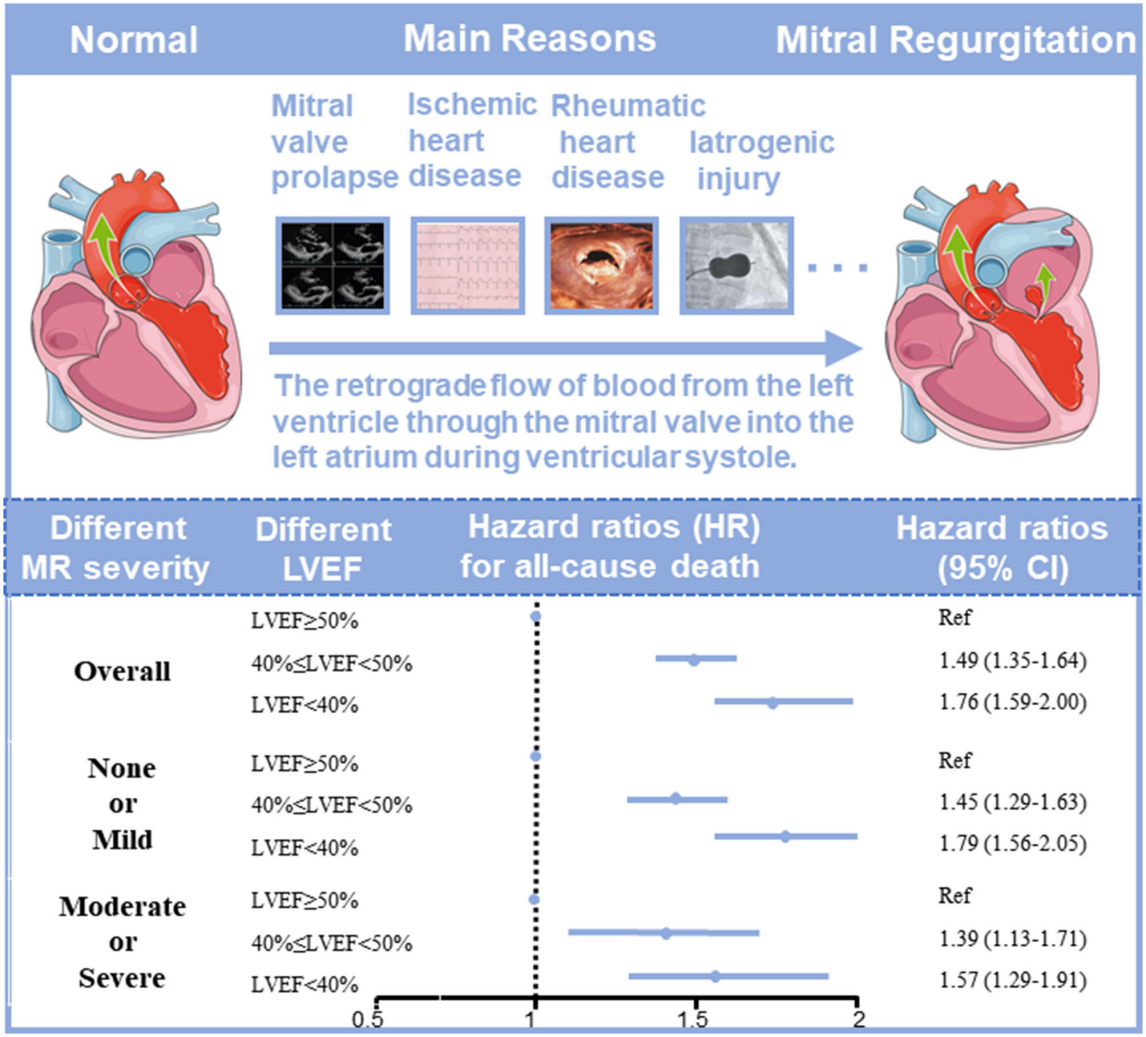
The association between left ventricular ejection fraction (LVEF) and long-term prognosis in coronary artery disease (CAD) patients with different severities of mitral regurgitation (MR).

**Figure 3:**
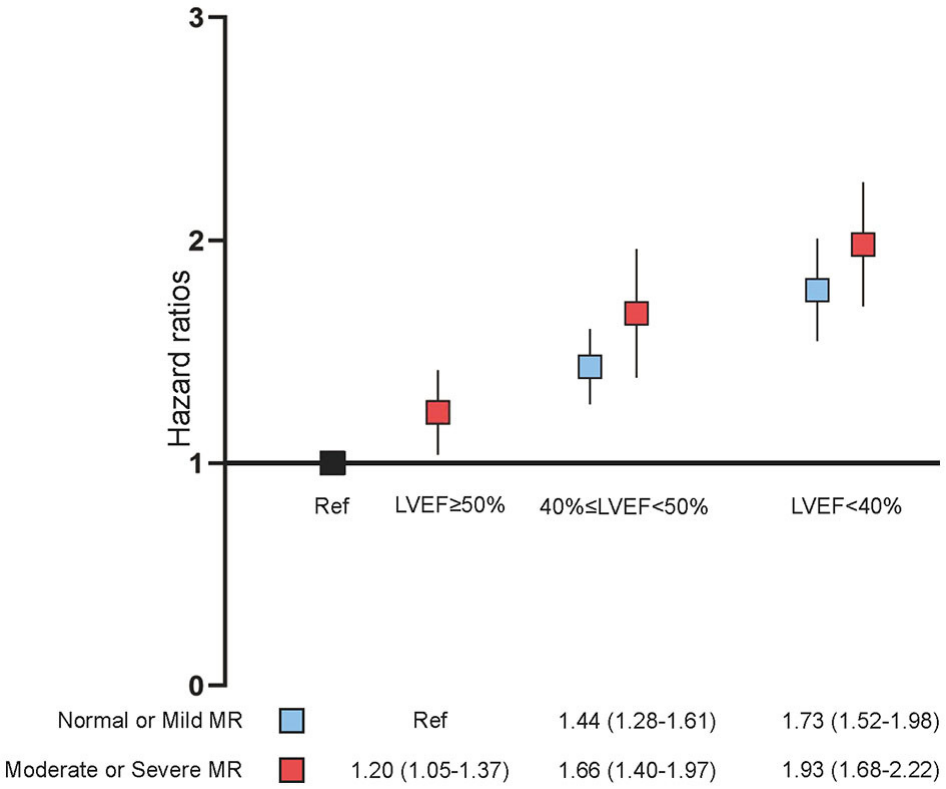
Multivariate Cox proportional analyses. Hazard ratios (HR) for all-cause death (95% CI) adjusted for age, gender, hypertension, diabetes mellitus, acute myocardial infarction, congestive heart failure, chronic kidney diseases, atrial fibrillation (AF), stroke, anemia, percutaneous coronary intervention (PCI), hyperlipidemia, angiotensin-converting enzyme inhibitor/angiotensin receptor blocker (ACEI/ARB), beta-blockers, calcium channel blocker (CCB), statins, antiplatelet, mineralocorticoid receptor antagonist (MRA), loop diuretics, and oral anticoagulants (OAC). MR, mitral regurgitation; LVEF, left ventricular ejection fraction.

In the univariate Cox proportional risk analysis, both the none or mild MR group and the moderate or severe MR group produced results that LVEF <40% was associated with an adverse event rate of all-cause death [Hazard ratio (HR): 2.38; 95% CI: 2.13–2.66, *p* < 0.001; HR: 2.23; 95% CI: 1.91–2.60, *p* < 0.001). After adjusting for age and gender, LVEF <40% remained significantly associated with all-cause death (HR: 2.27; 95% CI: 2.03–2.54, *p* < 0.001; HR: 2.27; 95% CI: 1.94–2.65, *p* < 0.001). On multivariable Cox proportional risk analysis, after adjustment for confusion factors (age, gender, HT, DM, AMI, CHF, CKD, AF, stroke, anemia, PCI, hyperlipidemia, ACEI/ARB, beta-blockers, CCB, statins, antiplatelet, MRA, loop diuretics, and OAC), LVEF <40% remained significantly associated with all-cause death regardless of MR severity—None or mild MR (HR: 1.79; 95% CI: 1.56–2.05, *p* < 0.001), and moderate or severe MR (HR: 1.57; 95% CI: 1.29–1.91, *p* < 0.001) (Table 2, Figure 2). Patients with LVEF <40% had a higher risk of death compared to patients with none or mild MR and LVEF ≥ 50%, after adjusting for confounding factors (Figure 3).

**Table 2:** Cox proportional hazard ratios (HR) for long-term all-cause mortality.

**Different base crowd**	**Different LVEF**	**Hazard ratios (95% CI), *P*-value**			***P* for** **interaction**
		Model 1^*^	**Model 2^$^**	**Model 3^§^**	
**None or Mild MR**	LVEF ≥ 50%	Ref	Ref	Ref	0.29
	40% ≤ LVEF <50%	1.65 (1.49–1.82), <0.001	1.65 (1.50–1.83), <0.001	1.45 (1.29–1.63), <0.001	
	LVEF <40%	2.38 (2.13–2.66), <0.001	2.27 (2.03–2.54), <0.001	1.79 (1.56–2.05), <0.001	
**Moderate or Severe MR**	LVEF ≥ 50%	Ref	Ref	Ref	
	40% ≤ LVEF <50%	1.56 (1.30–1.88), <0.001	1.55 (1.29–1.86), <0.001	1.39 (1.13–1.71), 0.002	
	LVEF <40%	2.23 (1.91–2.60), <0.001	2.27 (1.94–2.65), <0.001	1.57 (1.29–1.91), <0.001	

* *Unadjusted.*

$ *adjusted for age and gender.*

§ *adjusted for age, gender, hypertension, diabetes mellitus, acute myocardial infarction, congestive heart failure, chronic kidney diseases, atrial fibrillation, stroke, anemia, percutaneous coronary intervention, hyperlipidemia, angiotensin-converting enzyme inhibitor/angiotensin receptor blocker, beta-blockers, calcium channel blocker, statins, antiplatelet, mineralocorticoid receptor antagonist, loop diuretics, and oral anticoagulants. MR, mitral regurgitation; LVEF, left ventricular ejection fraction*.

## Discussion

This study is the first largest cohort study to evaluate the association between LVEF and long-term prognosis in CAD patients with different MR severities. In our study, low LVEF in the patient with none and mild MR increased the mortality risk by four-fifths compared to the patient with normal LVEF. In the patient with moderate and severe MR, this risk remained nearly three-fifths. Our study suggests that LVEF remains a reliable assessment of prognosis in patients with variable degrees of MR.

MR is a growing public health burden, whose prevalence increases with increasing age, and the incidence of MR will increase due to population aging and growth (13). MR generally progresses insidiously, and causes left-ventricular overload and dysfunction due to the heart compensating for increasing regurgitant volume by left-atrial enlargement. LVEF, the cornerstone of contemporary clinical practice, is defined as the stroke volume indexed to the end-diastolic volume (EDV). It is one of the most important measured variables in clinical practice and regularly used by clinicians to describe systolic function and contractility (13), guiding the therapies and clinical decision of a serious of cardiovascular disease (13, 14). Especially in patients with MR, LVEF is particularly important as an important prognostic factor in the evaluation of optimal timing for surgery (15–18). However, MR being the most common valvular abnormality worldwide in patients with CAD, it is caused by retrograde flow of blood from the left ventricle (LV) through the mitral valve into the left atrium (LA) during cardiac systole (19–21), and contributes to the confounding effect of MR volume (22). Therefore, the assessment of cardiac function in MR patients using LVEF may underestimate the degree of intrinsic myocardial systolic dysfunction. However, it remains controversial whether LVEF is an accurate assessment of the prognosis of patients with MR. LVEF is a cornerstone of contemporary clinical practice, guiding the use of treatments and interventions for a range of cardiovascular diseases. However, the value of LVEF in guiding patients with MR remains unclear. Rosenhek et al. (23) suggested that LVEF may remain in the normal range for a long time, which makes LVEF not an accurate assessment of cardiac function in patients with MR. Similarly, in patients with significant primary MR, cardiac magnetic resonance study recently showed that LV dilatation generally occurs in the mid apical section of the ventricle and only later, at an advanced stage of disease process, occurs at the LV base (24). Therefore, a number of studies have concluded that LVEF does not accurately assess the prognosis of patients with MR and have begun to try to find new and more accurate metrics (25, 26). However, Enriquez-Sarano et al. (15) concluded that among the predictors of mortality after mitral valve closure insufficiency surgery, preoperative echocardiographic LVEF remains the best predictor of late survival. Other studies have similarly found that low preoperative LVEF (<60%) predicts postoperative LV dysfunction and is independently associated with increased postoperative mortality (16, 27). This is consistent with our study, which confirms that LVEF remains consistently effective for patient prognosis, regardless of the degree of regurgitation in patients with mitral valve insufficiency. Furthermore, in patients with moderate to severe MR, a decrease in LVEF also suggests a poor prognosis.

The following mechanism may explain why the overestimated LVEF can remain effective in indicating prognosis. Although LV dysfunction may be hidden behind normal LVEF due to altered loading conditions (28), MR causes LV and atria to expand due to volume overload and increased preload, resulting in a series of compensatory myocardial remodeling, which may lead to irreversible depression of ventricular and atrial function, and LV dilation increases ventricular wall stress leading to deterioration of LV function (29). Subsequent LV dilatation in turn affects mitral leaflet engagement, and LV systolic dysfunction reduces the strength of mitral valve closure, leading to worsening of MR and further worsening LV dysfunction (30). Finally, over time, patients with severe MR develop irreversible impairment of LV systolic function (31) and exhibit a progressive decrease in LVEF.

Our study found that LVEF remains valid for assessing the prognosis of patients with MR, and lower LVEF value is associated with worse prognosis. Patients with MR should be actively followed up with echocardiography to assess the prognosis and adjust the treatment plan and means by focusing on the patient's LVEF. Strict LVEF monitoring should be performed in patients with normal LVEF in order to predict the patient's prognosis and propose reasonable therapeutic measures to improve the prognosis of MR patients in a timely manner. The prognosis of patients with moderate to severe MR with decreased LVEF should be actively improved by mitral valve surgery, which is the only way to improve left ventricular systolic dysfunction due to MR (32).

## Limitations

There are several limitations in our study. Firstly, the data was extracted from a single-center retrospective study, which hampered us from controlling a variety of confounders in analyses, whereas sizeable data extracted from medical records were allowed to control a variety of confounders and selection bias in analyses. Secondly, there existed population selection bias, and we lacked patients from primary hospitals. However, more than half of our population came from non-teaching hospitals and community hospitals, in urban and rural areas. Third, we lacked regular monitoring of dynamic changes in LVEF, which may be more important. However, the ultrasound on admission was performed by professional cardiac ultrasound experts with a small measurement bias. We cannot exclude the influence of other confounding factors on the results, including coronary artery bypass grafting (CABG) following CAG, the number of diseased vessels, and the incidence of full revascularization. The above variables are very meaningful for the analysis and interpretation of the results, and we will further collect and analyze the above variables in future studies. Finally, information about cause-specific death was not available in this study, and it is difficult to examine the significant correlation between LVEF and cause-specific death.

## Conclusions

LVEF is a reliable prognostic index in CAD patients regardless of MR severity, and helpful for risk stratification. Reduced LVEF is associated with poor outcome in CAD patients with MR. Therefore, follow-up cardiac ultrasound for these patients is highly warranted.

## Data Availability Statement

The original contributions presented in the study are included in the article/Supplementary Files, further inquiries can be directed to the corresponding authors.

## Ethics Statement

The studies involving human participants were reviewed and approved by Guangdong Provincial People's Hospital Ethics Committee. Written informed consent for participation was not required for this study in accordance with the national legislation and the institutional requirements.

## Author Contributions

YL, JL, NT, and JC: research idea and study design. QL, YZ, HH, WC, SS, SC, BW, WL, ZH, and ZL: data acquisition. JL and YL: data analysis/interpretation. SC and QL: statistical analysis. YL, JL, and JC: supervision and mentorship. All authors contributed important intellectual content during manuscript drafting or revision and accepts accountability for the overall work by ensuring that questions pertaining to the accuracy or integrity of any portion of the work are appropriately investigated and resolved. All authors contributed to the article and approved the submitted version.

## Funding

This study was supported by grants from the National Science Foundation of China (Nos. 81970311 and 82070360), Study on the function and mechanism of the potential target for early warning of cardiorenal syndrome after acute myocardial infarction based on transformism (DFJH201919), Beijing Lisheng Cardiovascular Health Foundation (LHJJ20141751), and Clinical Medicine Research Fund of Guangdong Province (2019ZX01). The funders had no role in the study design, data collection and analysis, decision to publish, or preparation of the manuscript.

## Conflict of Interest

The authors declare that the research was conducted in the absence of any commercial or financial relationships that could be construed as a potential conflict of interest.

## Publisher's Note

All claims expressed in this article are solely those of the authors and do not necessarily represent those of their affiliated organizations, or those of the publisher, the editors and the reviewers. Any product that may be evaluated in this article, or claim that may be made by its manufacturer, is not guaranteed or endorsed by the publisher.

## Supplementary Material

Supplementary Figure 1 Flow chart of the study population. CAD, coronary artery disease; CAG, coronary arteriography; LVEF, left ventricular ejection fraction; MR, mitral regurgitation.
