# Supplementary figures and images for: Are There Any Differences in the Prognostic Value of Left Ventricular Ejection Fraction in Coronary Artery Disease Patients With or Without Moderate and Severe Mitral Regurgitation?
Source: Front Cardiovasc Med. 2022 Mar 4;9:799253. doi: 10.3389/fcvm.2022.799253 (PMC8930921; doi:10.3389/fcvm.2022.799253)

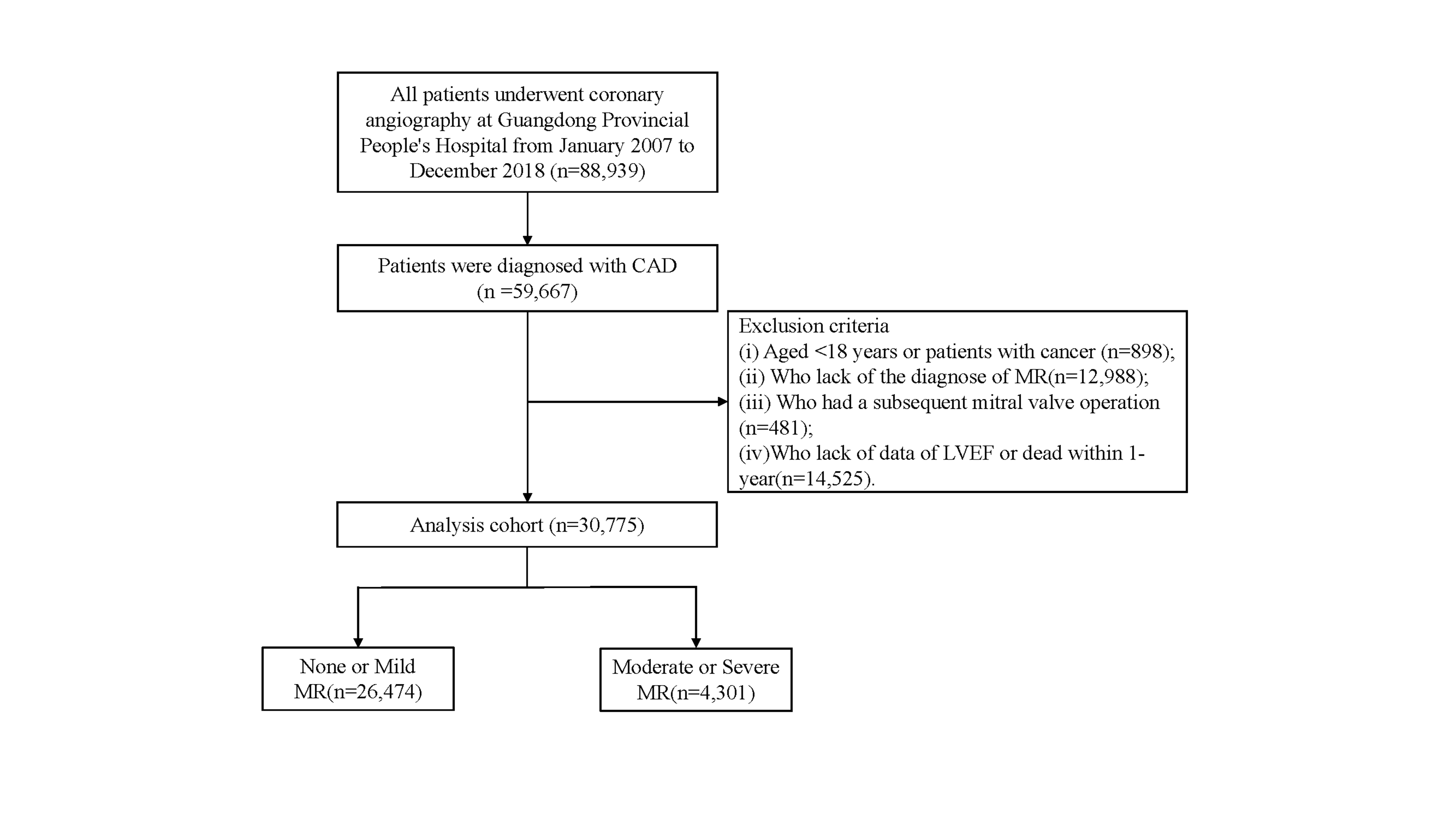

Supplement: Supplementary Figure 1 — Flow chart of the study population. CAD, coronary artery disease; CAG, coronary arteriography; LVEF, left ventricular ejection fraction; MR, mitral regurgitation. [file Image_1.TIF]
